# 836. Significant Elevations in CD4^+^ Count, Post-treatment of Chronic Hepatitis C (HCV) with Direct Acting Antivirals (DAAs), in Human Immune Deficiency, HIV/HCV Co-infected Patients with Prolonged CD4+ lymphocytopenia, despite undetectable HIV RNA

**DOI:** 10.1093/ofid/ofab466.1032

**Published:** 2021-12-04

**Authors:** Ryan Kuhn, Carolyn Green, Suganthini Krishnan Natesan

**Affiliations:** 1 John D. Dingell VA Medical Center, Detroit, Michigan; 2 John D Dingell VAMC/Wayne State University, Detroit, Michigan

## Abstract

**Background:**

Introduction of DAAs has revolutionized HCV therapy. Treatment of HIV/HCV co-infected patients is challenging and HCV treatment data on this group of patients are limited. **Aim: T**o review pre-and post-DAA treatment parameters, identify measures to improve recruitment and improve quality of care in co-infected veterans. A QC/QI project.

**Methods:**

A retrospective chart review of HIV/HCV co-infected patients treated for HCV with DAA at Detroit VAMC was performed. All patients were on anti-retroviral treatment for HIV with undetectable viral loads. Pre-and post-DAA treatment parameters were compared in patients who completed 12 weeks of treatment. Drug interactions, SVR, CD4^**+**^ counts AST, ALT, albumin, INR, platelets, creatinine, alpha-fetoprotein (AFP), HCV RNA, and FIB-4 score were recorded. Pearson correlation coefficient was used for data analysis.

**Results:**

Out of 46 patients, 4 died and 20 were ineligible due to non-compliance, mental illness, or drug use; 22 eligible patients, who had well controlled HIV, received DAAs for 12 weeks. (Genotype was 1a in 14, 1b in 7 and 2b in 1 patient). Compliance rate was 100%, 21 patients were HCV treatment naïve, 1 treated with interferon in the past) and all 22 patients achieved SVR by 12 weeks (in 2 weeks), including patients (n=12) on long term opioids and/or mental health treatment. Among 10/24 patients who showed a significant increase in CD4+ (range > 100 to 400 within 6 months), 8 were cirrhotic and had received DAA + RIB therapy. HIV therapy regimen change to alafenamide combination was required in 7/22 patients, for renal dysfunction. There were decreases in AST/ALT, but no changes in FIB-4 score, platelets, albumin, creatinine, or AFP were noted.

**Conclusion:**

HIV/HCV Co-infected patients who received DAA + RIB had a significant increase in CD4^**+**^ lymphocyte counts (p< 0.05) (unlike interferon-based regimen). Chronic opioid use and mental health treatment were not a hindrance to successful therapy. The clinical impact of our findings on long-term complications including cirrhosis, hepatocellular carcinoma, and extra-hepatic manifestations of HCV remain to be seen. Recognition of positive predictive markers will delineate the cohort of co-infected veterans who would benefit from DAA therapy beyond HCV eradication.

**Disclosures:**

**All Authors**: No reported disclosures

